# Organic Light-Emitting Diode Based Fluorescence-Linked Immunosorbent Assay for SARS-CoV-2 Antibody Detection

**DOI:** 10.3390/bios12121125

**Published:** 2022-12-05

**Authors:** Cheng Lian, Dan Young, Richard E. Randall, Ifor D. W. Samuel

**Affiliations:** 1 Organic Semiconductor Centre, SUPA, School of Physics and Astronomy, University of St Andrews, St Andrews KY16 9SS, UK; 2 Centre for Biomolecular Sciences, School of Biology, University of St Andrews, St Andrews KY16 9ST, UK

**Keywords:** OLED, sensing, point-of-care testing, FLISA, organic semiconductor

## Abstract

Immunodiagnostics have been widely used in the detection of disease biomarkers. The conventional immunological tests in central laboratories require expensive equipment and, for non-specialists, the tests are technically demanding and time-consuming, which has prevented their use by the public. Thus, point-of-care tests (POCT), such as lateral flow immunoassays, are being, or have been, developed as more convenient and low-cost methods for immunodiagnostics. However, the sensitivity of such tests is often a concern. Here, a fluorescence-linked immunosorbent assay (FLISA) using organic light-emitting diodes (OLEDs) as excitation light sources was investigated as a way forward for the development of compact and sensitive POCTs. Phycoerythrin (PE) was selected as the fluorescent dye, and OLEDs were designed with different emission spectra. The leakage light of different OLEDs for exciting PE was then investigated to reduce the background noise and improve the sensitivity of the system. Finally, as proof-of-principle that OLED-based technology can be successfully further developed for POCT, antibodies to severe acute respiratory syndrome coronavirus 2 (SARS-CoV-2) in human serum was detected by OLED−FLISA.

## 1. Introduction

Immunodiagnostics have become a widely used method for diagnosis of diseases and monitoring their transmission [1,2]. Medical decisions are supported by testing for antibodies to specific diseases. However, conventional immunological tests are based in central laboratories, involving bulky and expensive equipment, long sample turnaround time, and trained professionals for performing the tests [3]. The cost and specialized nature of such tests limits their availability to patients in developing countries [4]. Even in developed countries, the time needed to send samples to labs causes delay. In recent years, there has been an increasing trend of moving immunological tests from central laboratories to point-of-care testing (POCT) [2]. POCT is designed to be carried out in the direct vicinity of patients. It is low-cost, simple to use, and can be performed in hospitals, doctors’ offices, or by patients themselves. A typical example of POCT is an immunoassay in a lateral flow device (LFD). A LFD can generate the result in a short time (typically less than 30 min) with only a few drops of sample such as blood, serum, urine, or saliva. Gold nanoparticles are often used as indicators in LFD so human eyes can visually inspect the test results [5]. This is attractive in terms of simplicity but can have limited sensitivity. Fluorescence can be detected with high sensitivity using instruments. Additionally, quantitative tests are required for monitoring the level of immunity in the long term. Therefore, it is desirable to develop a test that is sensitive, quantitative, compact, low-cost and easy to use. Here, we explore the use of organic light-emitting diodes (OLEDs) to make a simple fluorescence-based immunoassay.

The basic components of fluorescence sensing involve a light source, filter sets and a detector. The target analytes are labelled with dyes and then excited by the light, and the detector collects the fluorescence from the dyes to compare with the reference sample. It is important that the components for POCT need to be compact enough to match the size of the system. Hence, cumbersome optical components such as lasers, filtered lamps, photomultipliers, and spectrometers are not suitable for POCT. Compact light sources such as inorganic light-emitting diodes (LEDs) and OLEDs are considered suitable candidates for POCT. Several works using compact systems based on LEDs and OLEDs to detect disease biomarkers have been reported. LEDs were used in microfluidic devices for detecting myoglobin and CK-MB [6]. OLED-related works were also reported in detecting human serum albumin [7], immunoglobulin A antibody [8], immunoglobulin G antibody, human papillomavirus antibody [9], and other dye-conjugated antibodies [10,11]. In addition to disease biomarkers, some studies also evaluated the system sensitivity by detecting dyes [12,13,14,15,16,17]. It was demonstrated in our previous proof-of-principle work that OLEDs can be used for sensing dye-labelled DNA [18]. Titov et al. demonstrated sensing of dye-labelled DNA in a monolithic OLED−Organic photodiode matrix for the multiplexed test [19]. Compared to LEDs, OLEDs are more flexible in design due to their material properties and fabrication methods. OLEDs can be patterned on different substrates at a very thin total thickness [20]. The low processing temperature of OLED fabrication can potentially lead to low-cost and disposable devices for POCT [14,17]. Furthermore, the customizable pixel geometry of OLEDs is promising to be used in integrated multiplexed tests, enabling the detection of different diseases in a single chip [10,19].

Although OLEDs have attractive features for POCT, many aspects of OLEDs need to be tailored to fit the requirements of specific sensing systems and analytes. One of the challenges is the broad emission spectrum of OLEDs that causes the leakage of excitation light after filtering. Hence, rejecting the excitation light effectively and minimizing the noise of the sensing system can be crucial to achieve sensing with high sensitivity. Moreover, testing the actual disease biomarkers from the patients can be more realistic in reflecting the capability of OLEDs for POCT. Here, we demonstrate the sensing of antibodies to severe acute respiratory syndrome coronavirus 2 (SARS-CoV-2) from patient serum using an OLED-based system as a proof-of-principle test for sensing disease biomarkers. Sensing SARS-CoV-2 can be a good demonstration as there are various types of tests available on the market such as nucleic acid tests and serological tests. In view of the limited testing capacity caused by the rapid growth of the pandemic, it is desirable to have a sensitive, accurate, compact and convenient test for point-of-care use [21]. The test we developed was performed by using a fluorescence-linked immunosorbent assay (FLISA), which can immobilize the target analytes on the surface for fluorescence sensing. A comparison was made between OLEDs with different emission spectra as excitation light sources for the leakage light investigation. With the carefully developed OLEDs, desirable results of sensing the presence of antibodies to SARS-CoV-2 in diluted patient serum were achieved. The demonstration of OLED−FLISA for sensing disease antibodies is an important step towards exploring the use of OLEDs for fluorescence immunoassays. Our exploration of OLED structure and leakage light in sensing system can be useful for the future design of point-of-care fluorescence immunoassays.

## 2. Experimental Methods

We describe experimental methods and details (e.g., how OLEDs are made) in this section. As this article substantially concerns instrument development, we consider improving OLED design and system design, together with test measurements in the results section.

### 2.1. Photoluminescence Measurements

The photoluminescence excitation (PLE) and photoluminescence (PL) of phycoerythrin (PE) solutions were measured in a cuvette by a fluorimeter (FS5, Edinburgh Instruments; Edinburgh, UK). The PE was prepared at 100 nM in phosphate-buffered saline (PBS). For the PLE measurement, the detection wavelength was 680 nm. For the PL measurement, the excitation wavelength was 350 nm.

### 2.2. OLED Fabrication

OLEDs were fabricated by thermal evaporation (EvoVac, Angstrom Engineering Inc.; Kitchener, ON, Canada) at a base pressure of 3 × 10^−7^ mbar. The size of the OLEDs used in this work was 4 mm by 4 mm. For the rate of deposition, the Al anode was deposited at 3 Å/s; hole transport layer (HTL) was deposited at 0.6 Å/s; emission layer (EML), hole-blocking layer (HBL) and electron-blocking layer (EBL) were at 0.3 Å/s; electron transport layer (ETL), Ag cathode and capping layer were at 1 Å/s; The OLEDs were unloaded directly into a glove box with a nitrogen atmosphere and encapsulated with glass lids and epoxy glue.

### 2.3. OLED Characterization

The electroluminescence (EL) spectra of the OLEDs were taken by a spectrograph (MS125, Oriel; Irvine, CA, USA) connected to a charge-coupled device (CCD) camera (DV420-BU, Andor; Belfast, Northern Ireland). For OLED characterization, a source meter (Keithley 2400, Keithley Instrument, Solon, OH, USA) was used to scan OLED devices with constant voltage. The optical performance was measured with a multimeter (Keithley 2000, Keithley Instrument, Solon, OH, USA) and a calibrated Si photodiode.

### 2.4. Fluorescence Sensing System Based on OLED Excitation

For OLEDs driven under the pulse operation for fluorescence sensing, a pulse generator (HP 8114A, Keysight Technologies; Santa Rosa, CA, USA) was used to supply pulsed voltage with a width of 300 µs at 10 Hz. The fluorescence was detected by a silicon photodiode (PDA100A-EC, Thorlabs; Newton, NJ, USA) and read out by an oscilloscope (MSO 3014, Tektronix; Beaverton, OR, USA). The gain of the Si photodiode was set to be 70 dB. A 20.5 Ω resistor was connected in series for protection and to measure the current in the circuit. The voltage produced by the pulse generator was 100 V, which was distributed across OLED and resistor, resulting in a different voltage directly applied to the OLEDs due to the difference in the device structure.

The basic operation of the fluorescence sensing system is to compare the difference in fluorescence intensity between samples. The fluorescence of the reference samples was first measured under the excitation of OLEDs and collected by the photodiode, then the samples with target analytes were introduced and measured using the same system setup. By comparing the difference in fluorescence signal between the target sample and reference sample, the presence of the target analytes can be determined. The result obtained by oscilloscope was processed by a computer with a Python program. The program calculated the subtracted fluorescence signal by taking the difference between the fluorescence of target sample and reference sample, and then averaged for 800 data points. The testing time for each sample in this fluorescence sensing system is less than 30 s, which is fast enough to be used in POCT.

### 2.5. Fluorescence-Linked Immunosorbent Assay (FLISA)

The chambered slides (#154852PK, Lab-Tek; Hailsham, UK) were coated with mouse anti-V5-Tag antibody (#MCA1360, BioRad; Hercules, CA, USA), 10 µg/mL in PBS, with gentle agitation overnight at 4 °C. Then, the unbound antibody was removed, and the slides were blocked with PBS containing 5% *w*/*v* Bovine Serum Albumin (BSA) (#A2153, Sigma-Aldrich, Sofia, Bulgaria) and 0.1% *v*/*v* Tween 20 (P1379, Sigma, Sigma-Aldrich, Sofia, Bulgaria) at a volume of 1 mL/well for 1 h at room temperature. Slides were then incubated with a lysate of cells expressing V5-tagged SARS-CoV-2 Nucleoprotein in PBS containing 0.1% *v*/*v* Tween 20 (PBS-T), with gentle agitation, for 2 h at 4 °C. Unbound antigen was washed away with PBS-T, and the slides were blocked with PBS-T containing 5% *w*/*v* BSA and 10 µg/mL mouse anti-V5-Tag antibody containing PBS-TBV for 1 h at room temperature. Then, the blocking buffer was removed, and the slides were incubated with samples of sera from control and SARS-CoV-2-convalescent patients in a dilution of 1:80 or 1:160 in PBS-TBV, 0.5 mL/well, for 1 h at room temperature with gentle agitation. Again, the unbound antibody was washed away with PBS-T. The slides were incubated with phycoerythrin-conjugated donkey-anti-Human IgG antibody (ab102439, Abcam; Cambridge, UK) diluted 1:1000 in PBS-TBV, 0.5 mL/well for 1 h at room temperature with gentle agitation. The unbound antibody was washed away with PBS-T and well chambers were removed. Finally, the coverslips were mounted with Citifluor AF-1.

## 3. Results and Discussions

### 3.1. Phycoerythrin as Fluorescence Indicator

**Figure 1:**
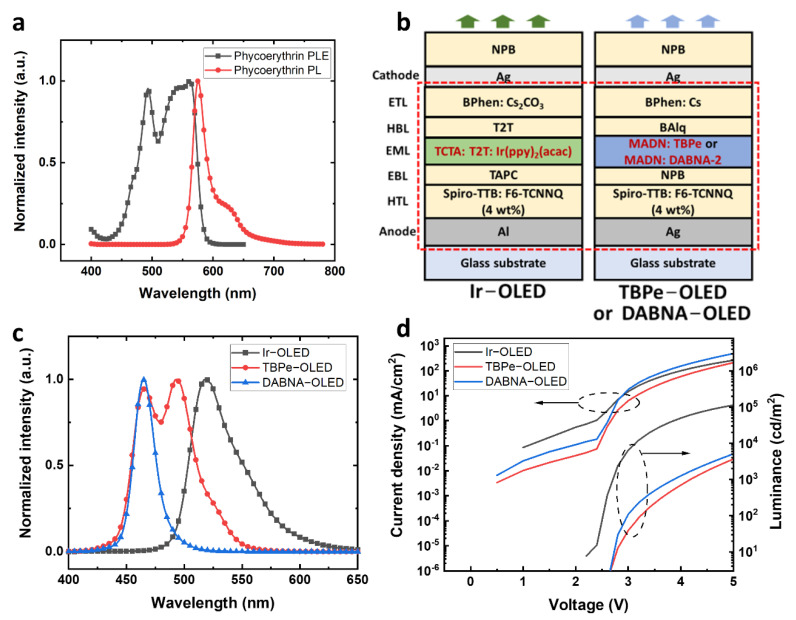
Phycoerythrin (PE) spectra and OLED information. (**a**) Photoluminescence excitation (PLE) and photoluminescence (PL) spectra of PE. (**b**) Device structures of OLEDs based on Ir(ppy)_2_(acac), TBPe, and DABNA-2 emitters. (**c**) Electroluminescence (EL) spectra of Ir−OLED, TBPe−OLED and DABNA−OLED. (**d**) *J*−*V*−*L* characteristics of Ir−OLED, TBPe−OLED and DABNA−OLED.

It is important to choose a suitable dye as the indicator to detect antibodies with fluorescence sensing. As OLEDs generally show broad emission spectra, reducing the long emission tail for lower background noise in fluorescence sensing is necessary [22]. The background noise can be reduced by carefully selecting the dyes with a large separation of absorption and emission, which allows OLEDs to excite the dyes efficiently while contributing less leakage light to disturb the detection of sample fluorescence. Although we have previously reported an approach to separate the absorption and emission by using Förster resonance energy transfer (FRET), it is not applicable in this test because the sensing target cannot be directly labelled to the disease biomarkers [18]. Therefore, using a dye with a good separation of absorption and emission spectra on its own is more desirable. Here, phycoerythrin (PE) was introduced as the dye for the test due to its high fluorescence quantum yield and good separation of absorption and emission spectra. PE has a high fluorescence quantum yield of 0.98 but poor photostability [23]. The photoluminescence excitation (PLE) and photoluminescence (PL) spectra of PE are shown in Figure 1a. PE has a broad absorption from 430 nm to 600 nm. The two dominant PLE peaks are around 495 nm and 560 nm, and a narrow emission peak can be found at 576 nm. Although the separation between the absorption and emission peaks of PE is only 16 nm, the excitation can still be very efficient at the wavelengths between 480 nm to 570 nm, where the PLE intensity is above 63% of the maximum value. The broad absorption of PE provides a wide range of suitable excitation wavelengths, allowing blue or green OLEDs to be used as excitation light sources. Therefore, PE was used as the fluorescence indicator in our fluorescence sensing system.

### 3.2. Development of OLEDs for PE Excitation

OLEDs with green or blue emissions were designed to excite the PE. A top-emitting structure was used for OLEDs to achieve narrower emission spectra and wavelength-tuning using the microcavity effect. Furthermore, doped charge transport layers were used for making p-i-n OLEDs with high conductivity and high brightness. PE can have an efficient excitation at green wavelengths but a smaller separation between the excitation and fluorescence compared to excitation at blue wavelengths. Here, a green OLED and two blue OLEDs based on different emitters were developed to deliver different excitation spectra. The green OLED, Ir−OLED, with a similar structure to our previous work was developed for exciting PE (Figure 1b) [18]. The emission layer (EML) of Ir−OLED was based on a phosphorescent emitter bis [2-(2-pyridinyl-*N*)phenyl-*C*](acetylacetonato)iridium(III) Ir(ppy)_2_(acac) doped in a co-host system formed by tris(4-carbazoyl-9-ylphenyl)amine (TCTA) and 2,4,6-tris(biphenyl-3-yl)-1,3,5-triazine (T2T) for higher conductivity and device efficiency. The overall structure of Ir−OLED is Al (80 nm)/2,2′,7,7′-tetra(*N*,*N*-di-*p*-tolyl)amino-9,9-spirobifluorene (Spiro-TTB): 2,2-(perfluoronaphthalene-2,6-diylidene)dimalononitrile (F6-TCNNQ) (4 wt%) (40 nm)/1,1-bis[(di-4-tolylamino)phenyl]cyclohexane (TAPC) (10 nm)/TCTA: T2T: Ir(ppy)_2_(acac) (0.46: 0.46: 0.08 in wt%) (20 nm)/T2T (10 nm)/4,7-diphenyl-1,10-phenanthroline (BPhen): Cs_2_CO_3_ (40 nm)/Ag (20 nm)/*N*,*N*′-di(1-naphthyl)-*N*,*N*′-diphenyl-(1,1′-biphenyl)-4,4′-diamine (NPB) (70 nm).

Moreover, PE can also be excited with blue OLEDs. One blue OLED design used an efficient and stable device structure with an EML of 2,5,8,11-Tetra-tert-butylperylene (TBPe) doped in 2-methyl-9,10-bis(naphthalen-2-yl)anthracene (MADN), noted as TBPe−OLED. The structure of TBPe−OLED was Ag (100 nm)/Spiro-TTB: F6-TCNNQ (4 wt%) (170 nm)/NPB (10 nm)/MADN: TBPe (1.5 wt%) (20 nm)/Bis(8-hydroxy-2-methylquinoline)-(4-phenylphenoxy)aluminum (BAlq) (10 nm)/BPhen: Cs (55 nm)/Ag (20 nm)/NPB (40 nm). The other blue OLED design used a multi-resonance blue emitter, DABNA-2, with a narrow emission [24]. The structure of the DABNA−OLED was Ag (100 nm)/Spiro-TTB: F6-TCNNQ (4 wt%) (140 nm)/NPB (10 nm)/MADN: DABNA-2 (1.5 wt%) (20 nm)/BAlq (10 nm)/BPhen: Cs (55 nm)/Ag (20 nm)/NPB (40 nm).

As shown in Figure 1c, the devices with Ir(ppy)_2_(acac), TBPe and DANBA-2 emitters have very different electroluminescence (EL) spectra. The Ir−OLED has a green emission peak at 519 nm with a full width at half maximum (FWHM) of 48 nm. The TBPe−OLED has a broad blue emission with a dominant peak at 495 nm and a secondary peak at 465 nm, and FWHM of 57 nm. The DABNA−OLED showed an emission peak at 465 nm with a FWHM of 21 nm, which is less than half of that of Ir−OLED and TBPe−OLED. The current density-voltage-luminance (*J*−*V*−*L*) characteristics of Ir−OLED, TBPe−OLED and DABNA−OLED are shown in Figure 1d. At 5 V, the Ir−OLED has a current density of 266 mA/cm^2^ with a brightness of around 113,000 cd/m^2^. The TBPe−OLED showed a current density of 213 mA/cm^2^ at 5 V with a brightness of 3663 cd/m^2^. The DABNA−OLED achieved 496 mA/cm^2^ at 5 V with a brightness of around 5100 cd/m^2^. The higher brightness of the Ir−OLED arises from the high efficiency of the phosphorescent emitter. As shown in Figure S1, the external quantum efficiency (EQE) of the Ir−OLED was higher compared to TBPe−OLED and DABNA−OLED. Additionally, human eyes are more sensitive to green, which makes the Ir−OLED have higher brightness. For the blue OLEDs, the DABNA−OLED has a brightness of about 40% higher than the TBPe−OLED at 5 V, which is an encouraging result as the emission peak of DABNA−OLED was at 465 nm. Although the Ir−OLED has a higher efficiency because of the phosphorescent emitter, the aim of using different OLEDs was to explore the influence of their spectra on exciting (and detecting) PE. Thus, the brightness achieved in all the green and blue OLEDs was enough for exciting PE.

### 3.3. Fluorescence Sensing System and OLED Leakage Light Management

**Figure 2:**
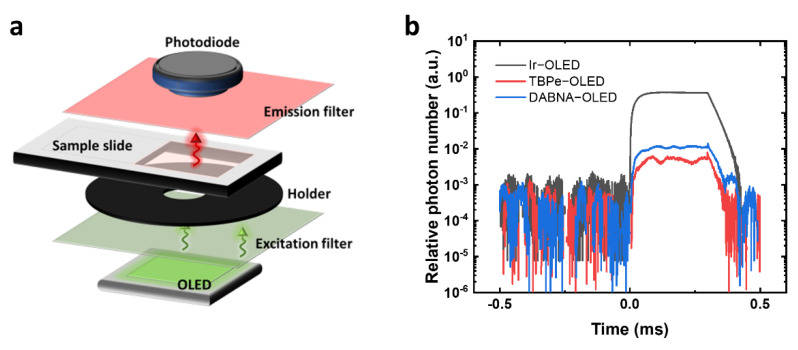
Fluorescence sensing setup. (**a**) Schematic diagram of the main components of the fluorescence sensing system. (**b**) Leakage light response of different OLEDs measured by the fluorescence sensing system in pulsed operation.

The fluorescence sensing system was first built to evaluate the excitation of PE using different OLEDs (Figure 2a). The main parts of the sensing system include an OLED as the excitation light source, an excitation filter to cut off the OLED emission tail, a black holder with an aperture of 8 mm in diameter, a transparent plastic slide for holding samples, an emission filter to cut off the excitation light, and a Si photodiode to detect the PE fluorescence. OLEDs were driven with square voltage pulses to reach high current density and achieve high brightness. A 100 V square voltage pulse was applied to the circuit and split between an OLED and a 20.5 Ω resistor. Thus, the voltage directly applied to OLEDs was different due to the difference in OLED resistance. As a result, the voltages directly applied to the Ir−OLED, the TBPe−OLED and the DABNA−OLED were 17.5 V, 18.2 V and 22.5 V, respectively. Figure S1b shows the lifetime evaluation of the Ir−OLED, TBPe−OLED and DABNA−OLED under voltage pulses. The Ir−OLED showed a high degradation compared to the TBPe−OLED and DABNA−OLED, which may be due to the use of T2T as cohost and HBL material. The TBPe−OLED and DABNA−OLED showed good stability with only 1% degradation in brightness after 6000 pulses. Therefore, Ir−OLED is not suitable for sensing applications because of its low stability. However, the Ir−OLED can still be used for leakage light evaluation in the sensing system.

In fluorescence sensing, different filter sets are needed to separate the excitation light and the sample fluorescence. Figure S2a−c show the spectral information of PE, the OLEDs and the corresponding filter transmittance used for different OLED devices. For the Ir−OLED, a short-pass filter (FES0550, Thorlabs) blocking light at wavelengths longer than 550 nm and a long-pass filter blocking light at wavelengths shorter than 575 nm (84−754, Edmund Optics; Barrington, NJ, USA) were used. The small separation in cut-off wavelengths between the excitation and emission filters can lead to high OLED leakage light. For the blue TBPe−OLED and DABNA−OLED, a short-pass filter blocking light at wavelengths longer than 500 nm (FESH0500, Thorlabs) and a long-pass filter blocking light at wavelengths shorter than 550 nm (FELH0550, Thorlabs) were used. Here, the leakage light was first calculated by multiplying the filter transmission and the OLED spectra. Then, a measurement using actual filter sets and OLEDs was done to determine the leakage light in our pulsed system. To calculate the leakage light, the emission spectra of the Ir−OLED, TBPe−OLED and DABNA−OLED were normalized so that their integral was 1 photon. The spectra of OLED leakage light were calculated according to the transmittance of filters (Figure S2d). The spectra were then integrated from 400 nm to 600 nm to calculate the fraction of leakage light. As a result, the Ir−OLED was estimated to have the highest leakage light among the three devices. A sharp peak of leakage light appeared at 540 nm, which can be attributed to the close cut-off wavelength of the filter sets used for Ir−OLED. The calculated leakage light for the TBPe−OLED was slightly lower than that of the DABNA−OLED. After the calculation, the actual leakage light in the fluorescence sensing system was measured by driving the OLEDs in pulsed mode and detecting the light intensity with the silicon photodiode after passing through the filter sets. The time response of the relative photon number shows that the leakage light for the Ir−OLED was at least 30 times higher than that of the TBPe−OLED and DABNA−OLED (Figure 2b), which makes the Ir−OLED difficult to be used in fluorescence sensing at low analyte concentration as it can increase the background of the detection. A high leakage light will overwhelm the fluorescence of the dye, and fluctuations in the leakage light then limit the sensitivity. The DABNA−OLED and TBPe−OLED have much lower leakage light, with the TBPe−OLED having around half the leakage light of the DABNA−OLED. According to the calculated results, the leakage light of the TBPe−OLED is similar to that of the DABNA−OLED. The difference between the calculated and actual leakage light is mainly due to the different pulse voltages at the OLEDs.

### 3.4. Evaluation of OLEDs for PE Excitation

**Figure 3:**
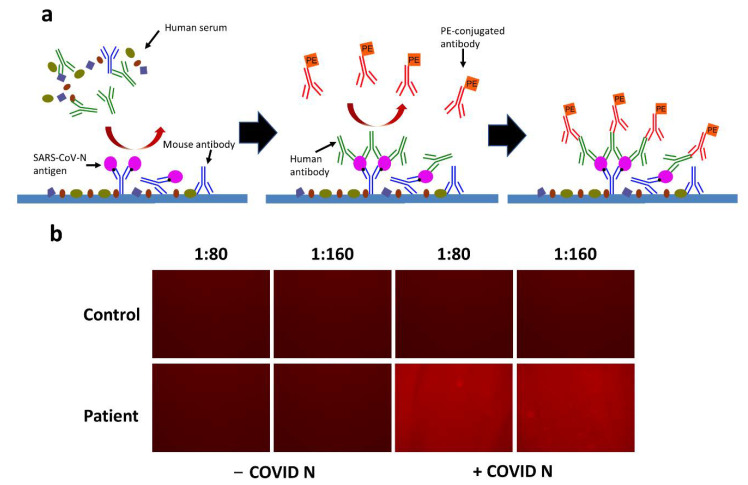
Demonstration of FLISA for sensing antibodies to SARS-CoV-2. (**a**) Schematic diagram of FLISA for sensing antibodies to SARS-CoV-2. (**b**) Photos of PE fluorescence in a fluorescence microscope. The human serum was diluted in 1:80 and 1:160. Comparisons were made between control and patient groups or non-expressed SARS-CoV-N (- −COVID N) and SARS-CoV-N (+COVID N) groups.

We next compared how the different OLEDs excite PE. Pure PE diluted in PBS was prepared at concentrations of 1.6 µM, 400 nM, 100 nM and 25 nM and put on a transparent plastic slide, with a coverslip placed on the top. To estimate the system sensitivity, signal-to-noise ratio (*SNR*) was used to compare the fluorescence signal level and the background noise level. It was defined as $SNR=\frac{S-B}{SD_{S-B}},$ where *S* is the raw fluorescence signal, *B* is the background signal, and *SD_S−B_* is the standard deviation of the fluorescence signal with background subtracted (S−B). The standard deviation of subtracted fluorescence was used in the calculation to take the fluctuation of fluorescence into account, avoiding the overestimation of *SNR* [18]. The *SD_S−B_* was obtained by calculating the standard deviation of averaged signal fluorescence in 80 µs (Figure S3). The limit of detection (LOD) was taken to be the concentration where *SNR* = 3, and the signal was considered to be detectable when *SNR* is larger than 3. Each of the PE samples was measured three times. The averaged fluorescence signal and *SNR* results under the excitation of the different OLEDs are shown in Figure S4. For the sample excited by the Ir−OLED, a very high average fluorescence signal and a high standard deviation across the three repetitions were observed (Figure S4a). The variation in average fluorescence is due to fluctuations of the high leakage light, making it difficult to achieve reliable sensing results. The unreliability of the Ir−OLED can also be found in SNR results (Figure 3b), where large fluctuations and negative values were observed at low PE concentrations. Therefore, the Ir−OLED is unsuitable for this fluorescence sensing system, especially at low analyte concentrations. The average fluorescence and SNR results were more stable for the TBPe−OLED and DABNA−OLED due to the suitable excitation wavelength and the good selection of filter cut-off wavelengths. The average fluorescence signal obtained with TBPe−OLED and DABNA−OLED was positive and with a small standard deviation across the three repetitions (Figure S4c,e). In terms of the *SNR*, it was found that both TBPe−OLED and DABNA−OLED can detect PE fluorescence at a lowest concentration of 100 nM. Assuming the volume of solution being excited was 0.7 µL, then the amount of PE detected at 100 nM was 0.07 picomoles. The DABNA−OLED generally had a higher *SNR* value than the TBPe−OLED but both blue OLEDs were considered suitable to explore as excitation light sources for FLISA.

### 3.5. OLED−FLISA for Sensing Antibodies to SARS-CoV-2

As introduced previously, FLISA can be a good method for detecting disease biomarkers. Here, human antibodies to SARS-CoV-2 were used as the analyte as they can be used to determine if a person has been previously infected by SARS-CoV-2. To perform FLISA, the plastic slides were prepared with a few procedures to immobilize and sense the analytes. Figure 3a shows the schematic diagram of FLISA for sensing antibodies to SARS-CoV-2 in a serological antibody test. First, the surface of the slide was bound with a saturated amount of mouse antibodies specific to the tag on the nucleocapsid protein SARS-CoV-N. The reagent containing blocking proteins was introduced to block the non-specific binding sites, and the excess materials were then washed away. Later, the cell lysate containing SARS-CoV-N was added to saturate the capture antibodies. After washing away the residual materials, the slide was ready to immobilize antibodies to SARS-CoV-2 specifically. Here, the diluted human serum was pre-incubated and introduced to the test. If the patient is infected or was previously infected by SARS-CoV-2, there should be target antibodies in the human serum that bind to the SARS-CoV-N. Again, the unbound materials were washed away. Finally, pre-incubated PE-conjugated antibodies (anti-human) that can bind to human antibodies were introduced. The human antibodies to SARS-CoV-2 can immobilize the PE-conjugated antibodies on the slide after another washing procedure. Finally, the fluorescence of PE can be detected under the OLED excitation if human antibodies to SARS-CoV-2 are present.

To test the FLISA procedure described above, diluted human serum (1:80 or 1:160) taken from a non-infected person (control) and coronavirus disease (COVID-19) infected person (patient) were prepared. The SARS-CoV-N was either expressed (+COVID N) or non-expressed (−COVID N), where only the expressed SARS-CoV-N can bind with the antibody. The FLISA was done with the stated procedures, and the fluorescence intensity was viewed under the fluorescence microscope (EVOS M5000, Thermal Fisher Scientific). As a result, no fluorescence was observed in the control group by comparing the brightness of the photos taken from the fluorescence microscope (Figure 3b). The absence of human antibodies to SARS-CoV-2 in the serum resulted in no immobilization of PE-conjugated antibodies. Hence, no fluorescence was shown. For the patient group, only the samples with expressed SARS-CoV-N showed fluorescence. The non-expressed SARS-CoV-N cannot bind to the capture antibodies, so the PE-conjugated antibodies were not immobilized either. It is not clear if there is any difference in the fluorescence intensity between 1:80 and 1:160 dilution of human serum according to the photo taken by the fluorescence microscope. Therefore, further quantitative detection with a photodiode is needed.

**Figure 4:**
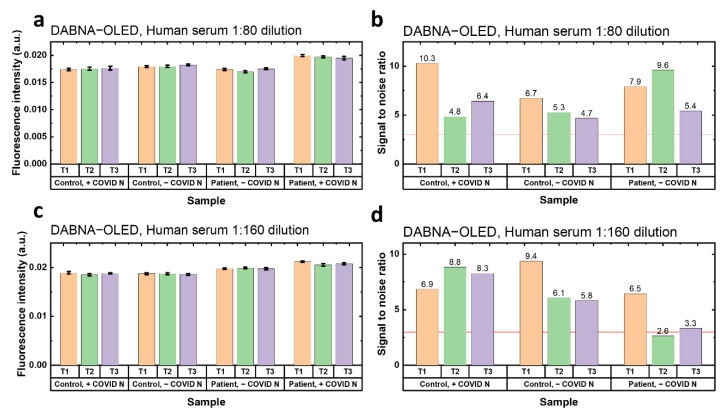
Results of OLED−FLISA (DABNA−OLED) for sensing antibodies to SARS-CoV-2. Each sample was measured three times. (**a**) Fluorescence intensity of samples tested with 1:80 dilution of human serum under the DABNA−OLED excitation. (**b**) *SNR* of samples tested with 1:80 dilution of human serum under the DABNA−OLED excitation. (**c**) Fluorescence intensity of samples tested with 1:160 dilution of human serum under the DABNA−OLED excitation. (**d**) *SNR* of samples tested with 1:160 dilution of human serum under the DABNA−OLED excitation. The error in the fluorescence intensity is shown in (**b**,**d**), but is hard to see because it is small compared to the overall fluorescence intensity.

Finally, the prepared FLISA samples were tested under the excitation of TBPe−OLED and DABNA−OLED. Each sample was tested three times for repetition (T1, T2 and T3). The fluorescence intensity in Figure 4 and Figure S5 is the raw fluorescence without background subtraction, which includes the fluorescence from the PE and OLED leakage light. Furthermore, the *SNR* was calculated here by taking the difference in fluorescence between the signal (patient, +COVID) and reference background (control, +COVID; control, −COVID; patient, −COVID). If the *SNR*s in the three reference backgrounds are all larger than 3, the antibodies to SARS-CoV-2 are considered detectable. The results of OLED−FLISA under the TBPe−OLED excitation are shown in Figure S5. For both 1:80 and 1:160 dilution of human serum, it was found that the fluorescence intensity of the positive group was only slightly higher than the reference group, and most *SNR*s were lower than 3, which means no antibodies were detected under the TBPe−OLED excitation. However, for the 1:80 dilution samples excited by the DABNA−OLED, higher fluorescence intensity can be seen from the group with patient serum and expressed SARS-CoV-N (Figure 4a). The *SNR*s of the samples in 1:80 human serum dilution were all higher than 3 (Figure 4b), which shows a successful detection of SARS-CoV-2 antibodies with DABNA−OLED excitation. The results of the samples in 1:160 human serum dilution also showed a good detection of SARS-CoV-2 antibodies, where the positive group showed the highest fluorescence intensity (Figure 4c). Additionally, two out of the three tests (T1 and T3) showed a *SNR* higher than 3 (Figure 4d). Therefore, it is also promising to sense the antibodies to SARS-CoV-2 with DABNA−OLED at 1:160 dilution of human serum.

It is desirable to know how OLED−FLISA compares to the commercial lateral flow antibody test. However, due to the difference in geometry and reagent, it is difficult to compare directly. Here, a rough comparison was made between our OLED−FLISA and a conventional antibody test. The same human serum samples used for OLED−FLISA without further dilution were tested in a commercial lateral flow antibody test kit (Panodyne COVID-19 antibody IgM/IgG rapid test kit). The results show that for the control test with non-infected human serum, only the control line was present (Figure S6a). For the test with the patient serum, a very faint line on the IgG can be observed (Figure S6b), which indicates a possible detection of IgG antibodies. However, a faint line is generally viewed as an invalid result in the lateral flow test. As the serum used in the successful OLED−FLISA was diluted at 1:80 dilution, it is estimated that OLED−FLISA is roughly 80 times more sensitive than this lateral flow antibody test in detecting antibodies to SARS-CoV-2.

Since there are other OLED-based systems for sensing disease biomarkers reported, it is necessary to compare our system to the reported works [8,9,10,11,25]. However, it is not easy to directly compare the system sensitivity of these works as there are many variations in dyes, detection equipment, and biomarkers. Thus, a comparison between the system designs and their advantages and disadvantages is shown here. The advantage of our system is that it uses a simple and efficient light source and detector for highly sensitive fluorescence sensing. In terms of the design of OLEDs, we used a p-i-n structure combined with a microcavity structure and a narrowband blue emitter, which is potentially more conductive and suitable as the light source for fluorescence sensing. In terms of detection, some of the works used CCD cameras as the detectors to achieve higher sensitivity and to obtain the fluorescence spectrum [7,8,10,11]. In contrast, we used an amplified silicon photodiode for detection. Although photodiodes cannot acquire spectral information, they are cheaper and more compact. Moreover, spectral information may be unnecessary as the integral intensity of the dyes is enough to determine the sensitivity. For the sensing platform, we consumed more reagents due to the larger surface area of the slide, which was 4 cm^2^. However, the actual sensing area on the slide illuminated by OLED was only 25 mm^2^. The geometry can be further improved by adopting the microfluidic systems used in other works for less reagent consumption and more convenient washing steps of FLISA [8,9,25]. The reported works have shown various attractive methods for detecting biomarkers in a more compact geometry. Thus, it is important to combine the advantages of other works and advance the design of OLEDs for FLISA.

## 4. Conclusions

In this work, a fluorescence-linked immunosorbent assay with OLEDs as excitation light source was demonstrated for detecting disease biomarkers. Phycoerythrin was used as the labelling dye due to its advantage of broad absorption and high quantum yield. OLEDs with different emitters were developed to investigate the influence of the spectra and leakage light on the sensing system. Blue OLEDs based on TBPe and DABNA-2 were found to be most suitable for PE excitation. Finally, a fluorescence-linked immunosorbent assay was used to sense antibodies to SARS-CoV-2 in human serum. The results show successful sensing of the antibodies using the OLED with DABNA-2 as the emitter. We performed a comparison which shows that the OLED−FLISA was able to detect antibodies at much lower concentrations than the commercial lateral flow antibody test. However, we did use a larger sample volume, something that could be improved in the future using microfluidics [14]. Although the OLED−FLISA we developed is not ready to be used in clinics, it is still compact and sensitive, and the system can be further improved for less reagent, smaller size, higher sensitivity, and multiplexed tests [6,19]. There is also a great potential for the current system to be developed as a quantitative measurement. Combining all the improvements mentioned, a sensitive and compact rapid test that can detect different disease biomarkers can be expected based on OLEDs. Overall, this successful demonstration of an OLED-based system for sensing disease biomarkers is promising for the future development of point-of-care testing.

## Data Availability

The data sets supporting this study are available at http://10.17630/95199603-a826-4de9-af3c-07f4f91326ee accessed on 23 November 2022.

## Supplementary Materials

The following supporting information can be downloaded at: https://www.mdpi.com/article/10.3390/bios12121125/s1, Figure S1. (a) EQE of Ir−OLED, TBPe−OLED and DABNA−OLED (assuming Lambertian emission). (b) Luminance change versus pulse number for Ir−OLED, TBPe−OLED and DABNA−OLED driven in pulsed operation at 17.5 V, 18.2 V and 22.5 V, respectively. The width of the voltage pulses was 300 µs at a repetition rate of 10 Hz; Figure S2. (a) PLE of PE, EL of Ir−OLED, the transmittance of 550 nm short-pass filter (FES0550, Thorlabs) and 575 nm long-pass filter (84-754, Edmund Optics). (b) PLE of PE, EL of TBPe−OLED, the transmittance of 500 nm short-pass filter (FESH0500, Thorlabs) and 550 nm long-pass filter (FELH0550, Thorlabs). (c) PLE of PE, EL of DABNA−OLED, the transmittance of 500 nm short-pass filter and 550 nm long-pass filter. (d) Calculated fraction of OLED leakage photon number after the filtration. The photon number produced by each OLED was normalized to 1; Figure S3. Example of the response of fluorescence signal detected by a silicon photodiode. The SD_S−B_ was obtained by calculating the standard deviation of the signal fluorescence (from 200 to 280 µs) with background subtracted; Figure S4. Evaluation of OLEDs for PE excitation. Three measurements with the same sample were done for each concentration. Mean and standard deviation of the fluorescence signal are shown. (a) Average fluorescence signal of PE under the excitation of Ir−OLED. (b) *SNR* calculated from the fluorescence sensing results of Ir−OLED. c) Fluorescence signal of PE under the excitation of TBPe−OLED. (d) *SNR* calculated from the fluorescence sensing results of TBPe−OLED. (e) Fluores-cence signal of PE under the excitation of DABNA−OLED. (f) *SNR* calculated from the fluorescence sensing results of DABNA−OLED; Figure S5. Results of OLED−FLISA (TBPe−OLED) for sensing antibody to SARS-CoV-2. Each sample was measured three times. (a) Fluorescence intensity of samples tested with 1:80 dilution of human serum under the TBPe−OLED excitation. (b) *SNR* of samples tested with 1:80 dilution of human serum under the TBPe−OLED excitation. (c) Fluorescence intensity of samples tested with 1:160 dilution of human serum under the TBPe−OLED excitation. (d) *SNR* of samples tested with 1:160 dilution of human serum under the TBPe−OLED excitation; Figure S6. Commercial antibody test with undiluted human serum. (a) Result of non-infected (control) human serum. No antibody was detected. (b) Result of infected patient serum (patient). A faint line of IgG was observed, but the line was too dim to be considered a valid test.
