# Supplementary material for: Organic Light-Emitting Diode Based Fluorescence-Linked Immunosorbent Assay for SARS-CoV-2 Antibody Detection
Source: Biosensors (Basel). 2022 Dec 5;12(12):1125. doi: 10.3390/bios12121125 (PMC9775261; doi:10.3390/bios12121125)
Supplement: Supplementary file 1 [file biosensors-12-01125-s001.zip › biosensors-2000227-supplementary.pdf]

biosensors

MDPI

Supporting information

Organic light-emitting diode-based fluorescence sensing system for SARS-CoV-2 antibody detection

Cheng Lian[1], Dan Young[2], Richard E. Randall[2], Ifor D. W. Samuel[1*]

1. Organic Semiconductor Centre, SUPA, School of Physics and Astronomy, University of St Andrews, St Andrews, KY16 9SS, UK
2. Centre for Biomolecular Sciences, School of Biology, University of St. Andrews, St. Andrews, KY16 9ST, UK

* Correspondence: idws@st-andrews.ac.uk

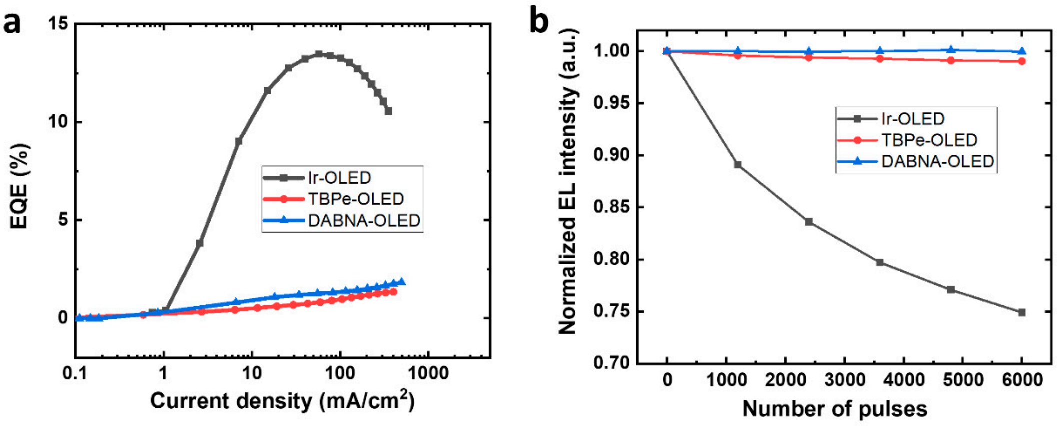

Figure S1. (a) EQE of Ir-OLED, TBPe-OLED and DABNA-OLED (assuming Lambertian emission). (b) Luminance change versus pulse number for Ir-OLED, TBPe-OLED and DABNA-OLED driven in pulsed operation at 17.5 V, 18.2 V and 22.5 V, respectively. The width of the voltage pulses was 300 μs at a repetition rate of 10 Hz.

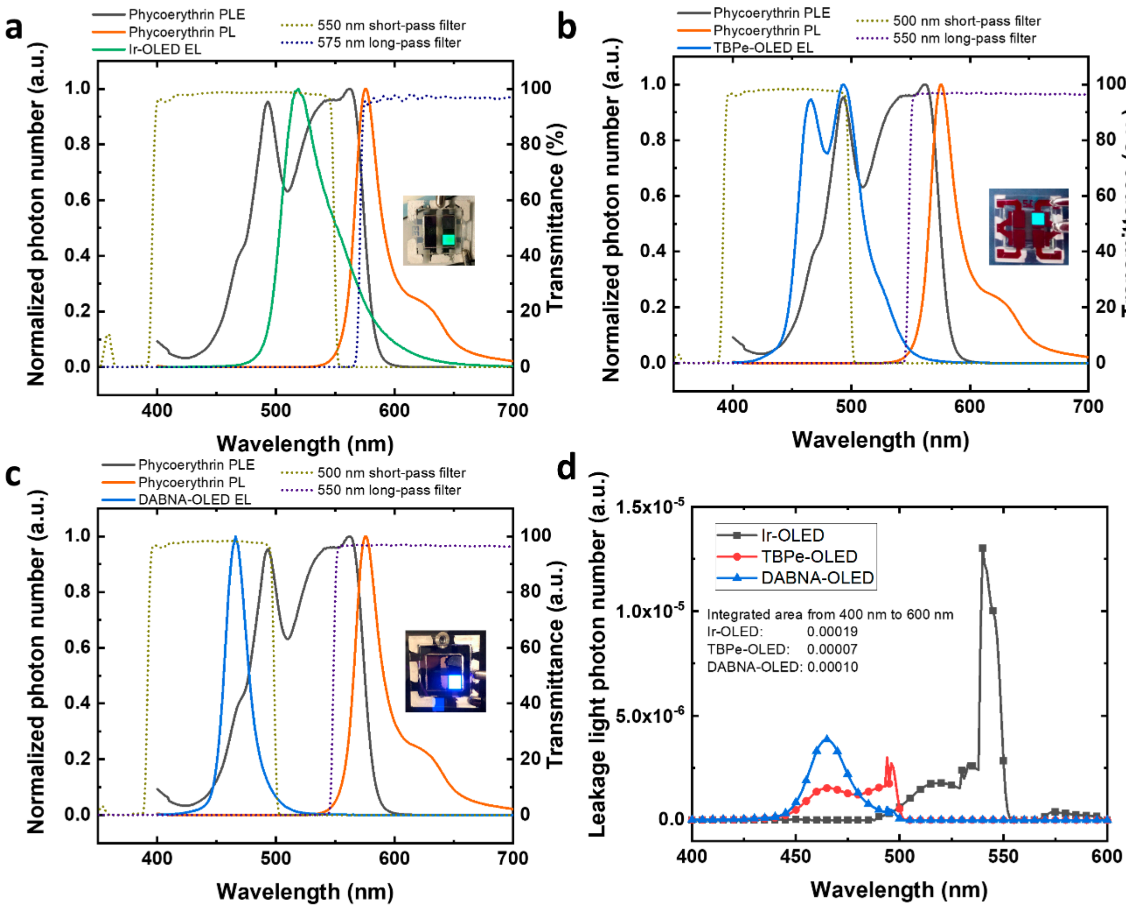

Figure S2. (a) PLE of PE, EL of Ir-OLED, the transmittance of 550 nm short-pass filter (FES0550, Thorlabs) and 575 nm long-pass filter (84-754, Edmund Optics). (b) PLE of PE, EL of TBPe-OLED, the transmittance of 500 nm short-pass filter (FESH0500, Thorlabs) and 550 nm long-pass filter (FELH0550, Thorlabs). (c) PLE of PE, EL of DABNA-OLED, the transmittance of 500 nm short-pass filter and 550 nm long-pass filter. (d) Calculated fraction of OLED leakage photon number after the filtration. The photon number produced by each OLED was normalized to 1.

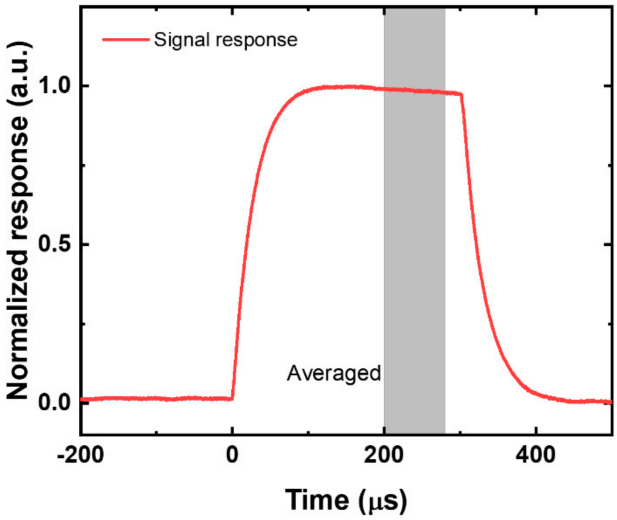

Figure S3. Example of the response of fluorescence signal detected by a silicon photodiode. The SD_{S-B} was obtained by calculating the standard deviation of the signal fluorescence (from 200 to 280 μs) with background subtracted.

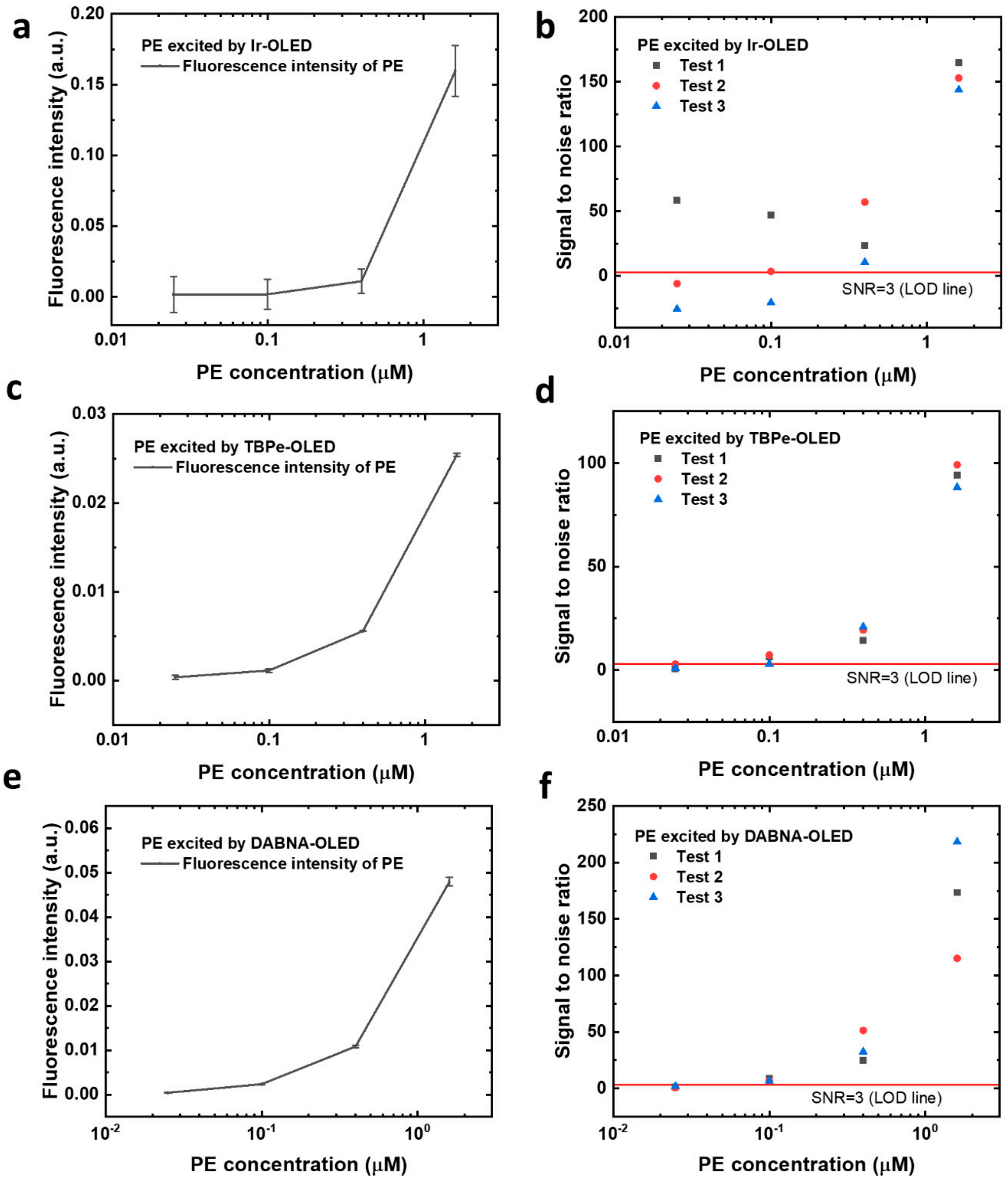

Figure S4. Evaluation of OLEDs for PE excitation. Three measurements with the same sample were done for each concentration. Mean and standard deviation of the fluorescence signal are shown. (a) Average fluorescence signal of PE under the excitation of Ir-OLED. (b) *SNR* calculated from the fluorescence sensing results of Ir-OLED. c) Fluorescence signal of PE under the excitation of TBPe-OLED. (d) *SNR* calculated from the fluorescence sensing results of TBPe-OLED. (e) Fluorescence signal of PE under the excitation of DABNA-OLED. f) *SNR* calculated from the fluorescence sensing results of DABNA-OLED.

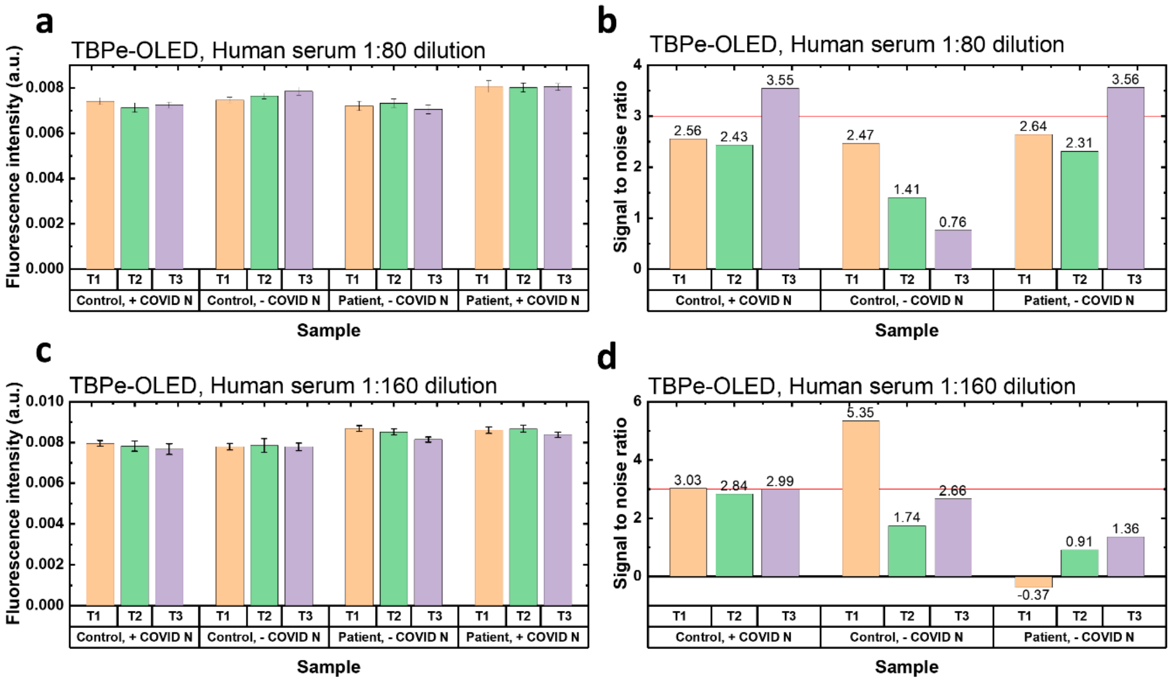

Figure S5. Results of OLED-FLISA (TBPe-OLED) for sensing antibody to SARS-CoV-2. Each sample was measured three times. (a) Fluorescence intensity of samples tested with 1:80 dilution of human serum under the TBPe-OLED excitation. (b) *SNR* of samples tested with 1:80 dilution of human serum under the TBPe-OLED excitation. (c) Fluorescence intensity of samples tested with 1:160 dilution of human serum under the TBPe-OLED excitation. (d) *SNR* of samples tested with 1:160 dilution of human serum under the TBPe-OLED excitation.

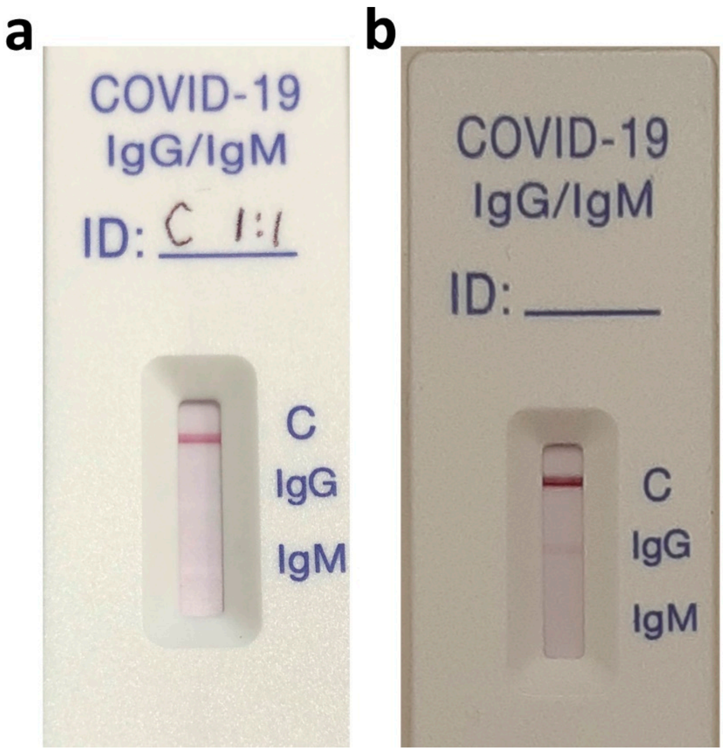

Figure S6. Commercial antibody test with undiluted human serum. (a) Result of non-infected (control) human serum. No antibody was detected. (b) Result of infected patient serum (patient). A faint line of IgG was observed, but the line was too dim to be considered a valid test.
